# Beyond Handgrip: Associations Between Trunk Strength, Gait Speed, Resting Metabolic Rate, and Muscle Mass in Brazilian Older Women with Probable Sarcopenia

**DOI:** 10.3390/ijerph23030338

**Published:** 2026-03-08

**Authors:** Lucas Ferreira de Souza Campos, Juliana de Alcantara Silva Fonseca, Ana Clara de Souza Oliveira, Guilherme Moreira, Leonardo de Souza Correa, Pedro Henrique de Almeida Louza, Ana Carolina Dutra Tavares, Luana Lopes de Souza, Raquel Carvalho Castiglione, Hércules Rezende Freitas, Silvio Rodrigues Marques Neto

**Affiliations:** 1 Programa de Pós-Graduação em Ciências da Atividade Física, Universidade Salgado de Oliveira (UNIVERSO), Niterói 24030-060, Brazil; lucasferreira653@hotmail.com; 2 Departamento de Ciências Biomédicas e Saúde, Instituto de Biologia Roberto Alcantara Gomes, Universidade do Estado do Rio de Janeiro (UERJ), Rio de Janeiro 28905-320, Brazil; ju.alcantarafonseca@gmail.com (J.d.A.S.F.); dutratavaresana@gmail.com (A.C.D.T.); luana.lsouzaf@gmail.com (L.L.d.S.); 3 Faculdade de Ciências Médicas (FCM), Universidade do Estado do Rio de Janeiro (UERJ), Rio de Janeiro 28905-320, Brazil; anaclaraoliveirat1@hotmail.com (A.C.d.S.O.); gmoreira10000@gmail.com (G.M.); leonardosousacorreax@gmail.com (L.d.S.C.); phalouza@gmail.com (P.H.d.A.L.); 4 Programa de Pós-Graduação em Biologia Humana Experimental (BHEx), Laboratório de Pesquisas Clínicas e Experimentais em Biologia Vascular (BioVasc), Universidade do Estado do Rio de Janeiro (UERJ), Rio de Janeiro 20550-013, Brazil; rccastiglione@gmail.com; 5 Health Informatics Laboratory (LabInfoS), Department of Integrated Medical Sciences, School of Medicine, State University of Rio de Janeiro, Rio de Janeiro 28905-320, Brazil; hercules.freitas@uerj.br; 6 Programa de Pós-Graduação em Fisiopatologia Clínica e Experimental (FISCLINEX), Laboratório de Pesquisas Clínicas e Experimentais em Biologia Vascular (BioVasc), Universidade do Estado do Rio de Janeiro (UERJ), Rio de Janeiro 20550-013, Brazil

**Keywords:** sarcopenia, muscle strength, physical performance, gait speed, functional mobility, aging, older women, sarcopenic obesity

## Abstract

**Highlights:**

**Public health relevance—How does this work relate to a public health issue?**

- Sarcopenia remains a significant public health challenge in aging populations, leading to reduced functional autonomy, metabolic health deterioration, and increased mortality risk among older women.
- Although handgrip strength is a standard diagnostic tool, it may not fully capture the functional status of muscle groups essential for postural control and locomotion, such as the trunk and lower extremities.

**Public health significance—Why is this work significant to public health?**

- This study demonstrates that assessments of lower limb and trunk strength provide critical clinical insights that go beyond the information provided by handgrip strength alone.
- The study revealed that maximal isometric hip extension (MIHE) is strongly correlated with resting metabolic rate (RMR), muscle mass, and key physical performance metrics, highlighting its value as a comprehensive health indicator.

**Public health implications—What are the key implications or messages for practitioners, policymakers, and/or researchers in public health?**

- Incorporating function-oriented evaluations, such as trunk strength and gait speed, can enhance the accuracy of sarcopenia screening in community-dwelling older women.
- Practitioners and policymakers should consider integrated diagnostic protocols that include both metabolic and functional markers to facilitate the early identification of individuals at risk of metabolic and physical decline.

**Abstract:**

Sarcopenia is a complex condition marked by reductions in muscle strength, mass, and overall physical performance, which has significant consequences for functional autonomy and metabolic health in elderly women. This study aimed to examine the correlations between lower limb strength, functional capabilities, and metabolic indicators in community-dwelling older women categorized according to the European Working Group on Sarcopenia in Older People 2 (EWGSOP2) criteria. A total of thirty-eight women aged ≥ 60 years underwent assessments, including anthropometric, hemodynamic, and metabolic evaluations, along with functional tests such as handgrip strength, chair-rise test, gait speed, Timed Up-and-Go, and maximal isometric hip extension strength (MIHE). The criteria for probable sarcopenia were established using the handgrip thresholds set by the EWGSOP2. Women identified as having probable sarcopenia displayed markedly lower MIHE, diminished gait speed, inferior performance in chair-rise and Timed Up-and-Go tests, decreased muscle mass, and a lower resting metabolic rate than their non-sarcopenic counterparts. MIHE exhibited robust correlations with muscle mass, resting metabolic rate, and functional performance metrics. These results suggest that assessments of lower limb and trunk strength yield pertinent insights beyond handgrip strength alone. Function-oriented evaluations may improve sarcopenia screening and facilitate the identification of older women at risk of functional and metabolic deficiencies in community-based environments.

## 1. Introduction

Sarcopenia is a progressive skeletal muscle disorder characterized by reductions in muscle mass (MM), muscle strength (MS), and physical performance, leading to functional impairment, loss of independence, and an increased mortality risk in older adults [1]. With the aging of the global population, improving screening-related decision-making is increasingly important for informing preventive and therapeutic strategies that preserve mobility and autonomy.

In 2019, the European Working Group on Sarcopenia in Older People (EWGSOP2) undertook a comprehensive revision of its screening approach, placing significant emphasis on the assessment of low MS as the primary criterion for diagnosing sarcopenia, with this condition being evaluated mostly through the measurement of handgrip strength (HG) [2]. Although the HG is recognized for its practicality, cost-effectiveness, and extensive validation within clinical settings, it may not adequately represent the functional status of the various muscle groups responsible for essential activities, such as postural control and locomotion, particularly those located in the trunk and lower extremities [3,4]. Evidence indicates that strength deficits in the proximal and lower limb regions are often more closely associated with mobility limitations and dependence on activities of daily living than isolated upper limb weakness [3,4].

Although HG is widely recognized as a practical surrogate marker of overall muscle strength, emerging evidence suggests that its relationship with lower limb muscle performance and asymmetry may not be entirely interchangeable. McGrath et al. [5] demonstrated that leg extension power asymmetry was independently associated with recurrent falls and fractures, even after accounting for HG. Similarly, Ostolin et al. [6] reported significant associations between HG and isokinetic muscle function of both the elbow and knee extensors; however, these associations were not uniformly proportional across muscle groups. These findings indicate that upper limb strength may not fully capture lower limb neuromuscular performance, particularly in contexts related to mobility decline and fall risk.

Functional performance assessments serve as a comprehensive evaluation of essential aspects, such as neuromuscular coordination, balance, and overall mobility. The Timed Up and Go (TUG) test, for instance, effectively captures dynamic postural control and locomotor capacity and has been consistently associated with both sarcopenia and an increased risk of falls among older adults [7,8,9]. Similarly, gait speed (GS) has been described as a functional vital sign because of its strong association with disability and overall survival rates. [7,10]. Although GS is recognized by the EWGSOP2 as a pertinent marker of physical performance [2], it reflects the coordinated function of multiple physiological systems, including lower-limb force production, postural stabilization, and cardiovascular fitness, supporting its role as a clinically relevant marker of functional status within the context of sarcopenia assessment [7,11,12].

From a musculoskeletal standpoint, the strength of the trunk extensor muscles plays a pivotal role in maintaining center-of-mass control. Trunk MS deficiency has been systematically linked to impaired balance and subsequent reduction in functional performance in older adults [4]. Concurrently, functional assessments, such as the 30 s chair rise test (CRT), provide reliable indicators of lower body strength in older adults residing in community settings [8], while evaluations of hip musculature have demonstrated commendable test–retest reliability and clinical feasibility [13]. Importantly, the effectiveness of the diagnostic criteria for sarcopenia may vary across different populations, and models that integrate both MM and functional measures have shown enhanced predictive capabilities for mortality within specific clinical contexts [14].

This observation underscores the necessity of validation tailored to specific populations, particularly among older Brazilian women, who are known to experience high rates of functional decline [15]. Furthermore, metabolic impairments frequently coexist with the deterioration of MM, contributing to alterations in the resting metabolic rate (RMR) and further exacerbating sarcopenia progression [1]. Nevertheless, relatively few studies have directly compared proximal lower limb strength and mobility-related tests with handgrip strength (HG) in relation to sarcopenia identification within this demographic. Consequently, the primary aim of the present study was to examine the associations between maximal isometric hip extension strength (MIHE), GS, and TUG performance and sarcopenia classification among older Brazilian women, as defined by the EWGSOP2 criteria, and to examine whether these functional measures differed between probable sarcopenic and non-sarcopenic participants [2].

Therefore, this study proposes an integrated physiological model combining MM, HG-defined classification, and proximal lower-limb strength (MIHE), GS, and RMR to support a multidimensional characterization of sarcopenia risk in older Brazilian women, addressing the limitations of single-marker screening approaches within a cross-sectional framework.

## 2. Materials and Methods

### 2.1. Study Design and Setting

This cross-sectional investigation was conducted by the Faculty of Medicine at the University of the State of Rio de Janeiro (UERJ), Cabo Frio Campus, as an integral component of a social initiative within a university extension program aimed at older women. The research was conducted at the facilities of the Secretariat for the Elderly (Secretaria da Melhor Idade), which operates under the Municipal Health Department of Cabo Frio, Rio de Janeiro, Brazil. This study was part of a more extensive study examining the functional capacity and metabolic factors associated with sarcopenia in elderly women.

The study was conducted at a community-based older adult center in Cabo Frio, which has approximately 200 registered participants who are regularly engaged in health promotion activities. All individuals attending the center during the data collection period were invited to participate voluntarily. A total of 47 older women agreed to participate in the study and were initially assessed. Of these, 38 completed all the evaluation procedures and were included in the final analysis. Nine participants were excluded because of incomplete assessments or inability to complete all functional testing procedures. No participant withdrew from the study after providing informed consent.

As this investigation was conducted as part of a community outreach and screening initiative, no prior sample size calculations were performed. The final sample size was determined based on the number of eligible participants available during the assessment period. A post hoc power estimation (α = 0.05; power = 0.80; two-tailed independent comparison) indicated that the final sample (n = 38) would be sufficient to detect large effect sizes (Cohen’s d ≈ 0.90 or greater). The magnitude of the observed between-group differences (d ranging from 0.96 to 1.33) exceeded this threshold, supporting the robustness of the primary group comparisons within this sample.

The eligibility criteria included physical independence, the ability to walk without assistive devices, and voluntary participation after receiving detailed information about the study procedures. The exclusion criteria comprised diagnosed neuromuscular or severe osteoarticular disorders, decompensated cardiovascular disease, cognitive impairment affecting task execution, and any condition contraindicating physical testing.

### 2.2. Participants

All participants underwent an initial health screening that included medical history, anthropometric assessment, and evaluation of hemodynamic and metabolic parameters. Information on comorbidities such as hypertension, diabetes mellitus, dyslipidemia, and hypothyroidism was obtained through self-report and confirmed, when available, through clinical documentation. Resting blood glucose and blood pressure were measured under standardized conditions after a 10 min seated rest, in accordance with the guidelines of the Brazilian Society of Cardiology.

Frailty and sarcopenia were assessed according to the EWGSOP2 framework [2], which integrates measures of muscle strength, quantity, and physical performance. Functional assessments included handgrip strength (HG), chair rise test (CRT), gait speed (GS), maximal isometric hip extension (MIHE), and timed up-and-go (TUG) test, as detailed below. The study was conducted in accordance with the Declaration of Helsinki and approved by the Research Ethics Committee (CEP) of the Pedro Ernesto University Hospital of the University of the State of Rio de Janeiro (HUPE/UERJ) (CAAE: 94655325.0.0000.5259). All participants provided written informed consent prior to the study.

This investigation was conducted within the context of a community-based social outreach initiative developed in partnership with the Faculty of Medical Sciences, aimed at providing clinical screening and functional assessment to community-dwelling older women. Therefore, the data collection protocol was structured primarily around a detailed clinical anamnesis and physical-functional evaluation, prioritizing health status, comorbidities, medication use, anthropometric measures, and neuromuscular performance indicators directly related to the objectives of the study.

Socioeconomic and educational variables were not systematically collected, as the initiative was designed with a clinical and health promotion focus rather than for comprehensive sociodemographic characterization. While this approach reflects the applied nature of the outreach setting, it may limit the broader contextual interpretation of the sample profile. Nevertheless, the absence of these variables did not affect the internal consistency of the strength-based stratification and functional metabolic analyses performed in the present study.

### 2.3. Sarcopenia Classification

#### Primary and Secondary Outcomes

The primary outcome of the study was the functional performance of the lower limbs and trunk, evaluated through maximal isometric hip extension strength (MIHE), gait speed (GS), chair rise test (CRT), and Timed Up-and-Go (TUG). These variables are indicative of the essential domains of mobility-related neuromuscular function. Secondary outcomes included muscle mass (MM) and resting metabolic rate (RMR), which are considered metabolic and body composition correlates of sarcopenia-related functional status. All outcomes were analyzed based on handgrip strength stratification (<16 kg vs. ≥16 kg), in accordance with the EWGSOP2 criteria for probable sarcopenia.

Sarcopenia was operationally defined and classified according to the EWGSOP2 criteria [2], which follow a stepwise diagnostic approach summarized as Find–Assess–Confirm–Severity (F–A–C–S) classification. Initial case finding was performed using the SARC-F questionnaire, which comprises five self-reported items related to strength, walking assistance, rising from a chair, stair climbing, and falls. Each item is scored from 0 to 2, yielding a total score of 0–10, with scores ≥4 indicating an increased risk of sarcopenia. The questionnaire was administered verbally by trained researchers using a standardized interview protocol.

Muscle strength (MS) was assessed as the primary diagnostic criterion using handgrip strength (HG) and chair rise tests (CRT). Probable sarcopenia was defined as reduced HG (<16 kg for women) or prolonged CRT (>15 s), in accordance with the EWGSOP2 thresholds [2]. In the present study, probable sarcopenia classification was based exclusively on the HG thresholds, in accordance with the EWGSOP2 recommendations. This operational definition was used for group stratification and did not imply diagnostic confirmation beyond the established criteria.

Confirmation of sarcopenia requires evidence of low muscle quantity. Muscle mass (MM) was assessed using multifrequency segmental bioelectrical impedance analysis (BIA) with the InBody 120 device (InBody Co., Seoul, Korea). All measurements were performed according to the standardized procedures provided by the manufacturer. Participants were evaluated in the morning after fasting for at least 4 h, avoiding vigorous physical activity for 24 h prior to testing, and voiding their bladder immediately before the assessment. Measurements were conducted with participants standing barefoot on the device electrodes, wearing light clothing, and without metallic accessories.

Appendicular skeletal muscle mass (ASM) was automatically estimated using the device’s proprietary algorithm and indexed to height squared (ASM/height^2^, kg/m^2^). A cutoff value of ≤6.0 kg/m^2^ for women was applied according to the EWGSOP2 criteria for low muscle quantity [2].

Resting metabolic rate (RMR) was also obtained from the same device and estimated using the predictive algorithm embedded in the InBody 120 system, which calculates energy expenditure based on measured body composition parameters, including fat-free mass, age, sex, height, and body weight. This estimation was derived from validated population-based equations incorporated into the device software. The values were expressed in kilocalories per day (kcal/day).

Severe sarcopenia was defined as the coexistence of low muscle strength and low muscle mass, combined with poor physical performance, characterized by a gait speed of <0.8 m/s or a TUG time of >12 s. This classification strategy is consistent with the EWGSOP2 recommendations and previous validation studies [2,8].

### 2.4. Functional Tests

All functional and strength assessments were conducted in a temperature-controlled laboratory environment (22–24 °C) by trained evaluators with experience in exercise physiology and geriatric assessment. Prior to testing, the participants performed a standardized warm-up consisting of 5 min of light walking, followed by dynamic lower limb mobility exercises. Each test was explained and demonstrated, and one familiarization trial was conducted.

#### 2.4.1. Handgrip Strength (HG)

Handgrip strength was assessed using a calibrated hydraulic hand dynamometer (Jamar^®^, Model 5030J1, Sammons Preston, Bolingbrook, IL, USA). The grip span was adjusted to fit each participant’s hand. The participants were seated with the shoulder adducted and neutrally rotated, elbow flexed at 90°, forearm in a neutral position, and wrist slightly extended. Three maximal voluntary contraction trials were performed for each hand, alternating sides, with 30 s rest intervals. The highest value obtained from the dominant hand was used for the statistical analysis. Values below 16 kg are indicative of probable sarcopenia in women, according to EWGSOP2 criteria [2].

#### 2.4.2. Chair Rise Test (CRT)

Lower limb functional strength was assessed using the five-times sit-to-stand test. Participants were seated on a standardized armless chair (seat height: approximately 43–45 cm) with their arms crossed over their chest. They were instructed to stand and sit five times as quickly as possible without using their upper limbs for support. Two trials were performed with a 1 min rest interval, and the shortest time was recorded [11].

#### 2.4.3. Gait Speed (GS)

Habitual GS was assessed over a central 6 m distance within an 8 m walkway, allowing 1 m for acceleration and 1 m for deceleration. The participants were instructed to walk at their usual comfortable pace. The tests were conducted on a flat, non-slip indoor surface. Two trials were performed, and the fastest time was used to calculate the GS (m/s). GS was calculated in meters per second, with values <0.8 m/s indicating reduced mobility [7,12].

#### 2.4.4. Maximal Isometric Hip Extension (MIHE)

Hip extensor strength was evaluated using a fixed Crown Dorsal Dynamometer (Filizola Ltda., São Paulo, SP, Brazil) with a maximum capacity of 200 kgf. The participants were positioned in a standardized standing posture with their trunks upright and stabilized. The dynamometer was attached at the level of the posterior distal thigh, and the participants were instructed to perform maximal voluntary isometric hip extension against fixed resistance. Three maximal contractions lasting 3–5 s were performed, with 60 s rest intervals between attempts. Verbal encouragement was provided to ensure a maximal effort. The highest value obtained was recorded and normalized to the body mass (N/kg) for analysis [13].

#### 2.4.5. Timed Up-and-Go Test (TUG)

The TUG test was performed using a standardized armless chair (seat height: approximately 43–45 cm). Participants were instructed to stand up, walk 3 m at their usual pace, turn around a marker, walk back, and sit down. The tests were conducted on a flat indoor surface. Two trials were performed, and the shortest time was used for the analysis. Values >12 s were considered indicative of impaired mobility and increased fall risk [2,14].

### 2.5. Statistical Methods

Data normality was assessed using the Shapiro–Wilk test. Descriptive statistics were used to characterize the samples. Continuous variables are presented as mean ± standard deviation (or median ± IQR, where appropriate), and categorical variables as absolute and relative frequencies. Between-group comparisons were conducted using independent Student’s *t*-tests or Mann–Whitney *U* tests as appropriate. In addition to *p*-values, 95% confidence intervals (CI) for mean differences were calculated to provide estimates of precision. Standardized effect sizes (Cohen’s d) were computed to quantify the magnitude of the between-group differences using pooled standard deviations. Effect sizes were interpreted according to conventional thresholds (0.2 = small, 0.5 = moderate, and 0.8 = large).

The associations between muscle strength, muscle mass, metabolic rate, and functional performance variables were examined using Pearson’s correlation coefficients. Statistical analyses were performed using GraphPad Prism^®^ (version 8.4.2, GraphPad Software, San Diego, CA, USA), with significance set at *p* < 0.05. Given the exploratory cross-sectional design and modest sample size, the analyses were primarily intended to examine associations rather than to establish predictive or diagnostic models. Therefore, the findings should be interpreted with caution, and larger studies are warranted to confirm these results.

## 3. Results

### 3.1. Anthropometric Characteristics of Participants

The comprehensive descriptive statistics are presented in Table 1.

Initially, 47 older women were recruited from a community-based health promotion program at the Secretariat for Elderly People in Cabo Frio, Brazil. Of these, 38 participants completed all the evaluation procedures and were included in the final analysis.

The participants were community-dwelling, physically independent, older women. Table 1 encapsulates the anthropometric profile of the cohort, which indicates an overall overweight status coupled with preserved peripheral girths. This pattern is characteristic of community-dwelling older Brazilian women and signifies the coexistence of central adiposity with relatively maintained limb circumference.

When stratified by handgrip strength, anthropometric variables related to body size and central adiposity exhibited minimal differentiation between groups, suggesting that overall body mass and fat distribution were not the primary factors associated with between-group strength differences in this sample.

Conversely, peripheral muscle-related circumferences (upper arm, forearm, and calf) were significantly greater in women with handgrip strength ≥16 kg, with large-to-very large effect sizes. This pattern indicates that limb girth is more closely associated with muscle strength than central adiposity markers, underscoring their relevance as morphological correlates of functional muscle capacity (Table 1).

These findings suggest that peripheral anthropometric indicators may be more closely aligned with muscle-related functional status than global adiposity measures in physically independent older adult women.

### 3.2. Hemodynamic Parameters, Disease History, and Lifestyle Habits

Table 2 presents the detailed values and proportions of the hemodynamic, clinical, and lifestyle characteristics of the participants.

The cohort generally exhibited a cardiometabolic profile characteristic of aging populations, marked by elevated systolic blood pressure and a moderate prevalence of chronic conditions. When stratified by handgrip strength, the measures of blood glucose and blood pressure did not demonstrate significant differentiation between the groups, indicating that these hemodynamic variables were not strongly associated with muscle strength classification in this sample.

Conversely, the resting heart rate was higher in the <16 kg group, with a moderate effect size, suggesting a potential association between lower muscle strength and altered cardiovascular or autonomic regulation. Although causal interpretation is not feasible, this pattern aligns with the concept that reduced muscular fitness may coexist with less favorable cardiovascular regulation in older adults. The medical history revealed comorbidities frequently associated with aging, with hypertension being the most prevalent condition, followed by diabetes mellitus, hypothyroidism, and dyslipidemia (Table 2).

Stratified descriptive analysis suggested a clustering of cardiometabolic comorbidities in the <16 kg group. Although the inferential comparison was not powered for categorical variables, this pattern was directionally consistent with the multidimensional vulnerability often observed in individuals with lower muscle strength.

Behavioral indicators suggested a tendency toward less favorable lifestyle patterns in the <16 kg group, including a higher prevalence of smoking and alcohol consumption. Given the limited sample size, these observations should be interpreted cautiously; however, they align with the broader notion that reduced muscle strength may be associated with adverse lifestyle factors in the aging population.

### 3.3. Physical Activity Modalities and Habits

The detailed distributions of the participants’ physical activity patterns are presented in Table 3.

Participants predominantly engaged in low-impact group-based physical activities, particularly water aerobics, gymnastics, and yoga. This profile reflects the typical structure of community programs for older women in Brazil, which emphasize social interaction, mobility maintenance, and flexibility rather than high-intensity neuromuscular conditioning.

Overall activity behavior was characterized by light-to-moderate perceived intensity and heterogeneous adherence duration, indicating that although the participants were regularly active, the training stimulus may have been insufficient to maximize lower limb and trunk strength adaptations.

This contextualizes the coexistence of functional variability despite regular participation in activities. Owing to the descriptive nature of the modality data and the limited statistical power for categorical stratification, no formal between-group comparisons were conducted.

### 3.4. Strength and Functional Performance

The comprehensive descriptive statistics are presented in Table 4.

The outcomes related to strength and functional performance according to the EWGSOP2 screening criteria are also detailed in Table 4. This investigation adhered to the EWGSOP2 F–A–C–S pathway [2]. Initially, the participants completed the SARC-F questionnaire, with a significant proportion exhibiting scores indicative of an elevated risk of sarcopenia, suggesting the presence of functional complaints consistent with early muscle impairment. Subsequently, handgrip strength (HG) and chair rise tests (CRT) were performed.

Overall, the HG and CRT values were close to the EWGSOP2 thresholds, indicating that the cohort comprised individuals with preserved to borderline muscle strength. This distribution substantiates the clinical rationale for stratifying the participants based on handgrip strength.

Additional functional assessments, including GS, TUG, and MIHE, exhibited moderate variability, reflecting the heterogeneous lower limb and trunk functional capacities of community-dwelling older women.

Although the mean gait speed and TUG values remained within the community reference ranges, the variability among participants suggested the presence of early functional decline in a subset of individuals. Collectively, these descriptive findings warrant subsequent stratified analyses based on handgrip-defined sarcopenia.

### 3.5. Primary Outcome: Functional Performance According to Handgrip Strength Classification

The numerical values are presented in Table 5. Participants were stratified according to handgrip-defined probable sarcopenia, comprising 17 women in the probable sarcopenia group (HG <16 kg) and 21 in the non-probable sarcopenia group (HG ≥ 16 kg). None of the patients in this cohort met the EWGSOP2 criteria for severe sarcopenia. When the participants were stratified based on probable sarcopenia, as defined by HG, consistent and substantial differences were observed across all functional performance measures (Table 5).

Women with probable sarcopenia exhibited poorer lower limb strength and mobility performance than their non-sarcopenic counterparts. Across functional outcomes, effect sizes were uniformly large to very large, indicating a significant functional distinction between the groups. Mobility-based tests relying on lower limb and trunk function (TUG and CRT) demonstrated the strongest largest between-group differences, followed by gait speed and maximal isometric hip extension strength. This pattern suggests that tasks requiring coordinated lower-limb force production and postural control demonstrated larger between-group differences according to strength-based classification.

The magnitude and consistency of these effects support the notion that handgrip-defined probable sarcopenia is associated with clinically relevant impairments in broader functional domains beyond upper-limb strength alone. As anticipated from the stratification criterion, handgrip strength exhibited the largest separation between groups.

Overall, these findings suggest that lower-limb–dependent functional tasks exhibited substantial between-group differences according to strength-defined classification, reinforcing their relevance as complementary functional indicators.

### 3.6. Secondary Outcomes: Muscle Mass and Resting Metabolic Rate According to Handgrip Strength Classification

Table 5 presents detailed descriptive statistics, revealing significant differences in metabolic and body composition parameters among the groups categorized by HG.

Notably, both MM and RMR were reduced in the probable sarcopenia group, with large effect sizes. This suggests that diminished HG is associated with concurrent decreases in muscle quantity and estimated metabolic expenditure, indicating an integrated structural–metabolic phenotype associated with lower muscle strength. The consistent direction and magnitude of these differences underscore the multidimensional nature of sarcopenia-related impairment in this cohort, extending beyond functional performance to include body composition and metabolic capacity of the participants.

These findings support the interpretation that handgrip-defined probable sarcopenia reflects broader physiological alterations rather than isolated upper limb weakness.

### 3.7. Correlation Analysis

**Figure 1:**
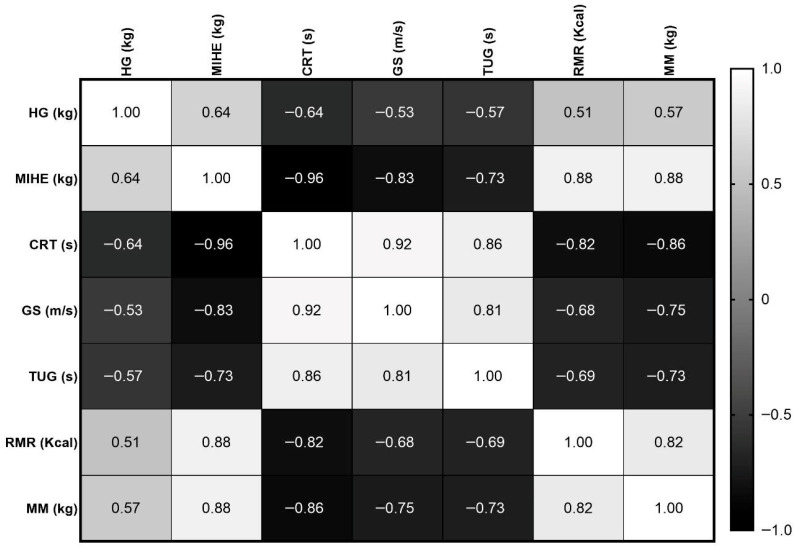
Correlation matrix heatmap illustrating the strength and direction of linear relationships among neuromuscular functions [handgrip strength (GP), maximal isometric hip extension (MIHE), chair rise test (CRT), gait speed (GS), Timed Up and Go (TUG), resting metabolic rate (RMR), and muscle mass (MM)]. Pearson’s correlation coefficients were color-coded, ranging from perfect positive (r = +1.0, white) to perfect negative correlations (r = −1.0, black).

The correlation patterns among strength, body composition, and metabolic and functional variables are shown in Figure 1.

Overall, the analyses revealed a highly coherent physiological network linking axial and lower limb strength, MM, RMR, and functional mobility in older women.

Trunk extension strength demonstrated robust positive associations with both RMR and MM, highlighting the strong coupling between axial muscle strength and lean tissue-related metabolic capacity. These high correlations indicate that individuals with greater trunk extensor strength also exhibit higher muscle quantity and resting energy expenditure, supporting the role of axial musculature as a central structural–metabolic component of the physical function.

Additionally, MIHE exhibited the strongest inverse association with chair rise performance, indicating that individuals with greater trunk extensor strength completed sit-to-stand transitions more rapidly. This finding reinforces the biomechanical relevance of trunk and hip extensors in transitional mobility tasks and suggests that axial strength is closely linked to functional lower-limb performance.

MM also showed a strong positive association with RMR, reinforcing the metabolic dependence of resting energy expenditure on the skeletal muscle mass. Conversely, lower muscle mass is consistently associated with poorer performance in mobility- and balance-related functional tests, indicating that reduced lean tissue is tightly coupled with diminished functional capacity.

Additionally, handgrip strength showed moderate associations with trunk strength, RMR, and MM, indicating that upper limb strength partially reflects overall muscular and metabolic status. However, the magnitude of these correlations was consistently lower than that observed for trunk extension strength, suggesting that handgrip strength is a more general but less function-specific indicator of the global muscle condition.

Furthermore, handgrip strength was moderately associated with functional performance measures, consistent with its established clinical utility as a global frailty and physical performance marker. Nevertheless, these relationships were weaker than those observed for axial strength, supporting the concept that trunk and lower limb musculature may directly underpin mobility-dependent function.

The performance-based functional tests (CRT, GS, and TUG) exhibited strong intercorrelations, reflecting the shared neuromuscular coordination, balance control, and lower limb force production required for these tasks. This clustering confirms that these measures capture overlapping domains of functional mobility and supports their combined use for characterizing functional impairment.

Collectively, these findings delineate a consistent pattern in which greater muscle strength and mass are associated with superior functional performance and higher metabolic rates, whereas reductions in these parameters are closely linked to mobility impairments. The strength and internal consistency of these correlations reinforce the interpretation that axial and lower limb strength constitute the central integrative components of the sarcopenia-related functional phenotype observed in this study population.

## 4. Discussion

The present observational pilot study demonstrated that maximal isometric hip extension strength (MIHE), gait speed (GS), and functional mobility measures provide clinically relevant information associated with sarcopenia classification in community-dwelling older Brazilian women, extending beyond the screening-related decision-making of handgrip strength (HG) alone. Women classified as having probable sarcopenia according to the EWGSOP2 criteria exhibited markedly lower MIHE, poorer functional performance, reduced muscle mass (MM), and a diminished resting metabolic rate (RMR). Importantly, the magnitude of these between-group differences was consistently large (Cohen’s d ranging from 0.96 to 1.33 for MIHE, GS, CRT, TUG, MM, and RMR, and 2.87 for HG), suggesting a substantial functional separation between strength-defined groups. However, because stratification was defined a priori by HG, these effect sizes should be interpreted as reflecting associative group separation within a screening framework, and not as evidence of diagnostic accuracy, predictive validity, or independent classification performance.

These effect sizes are comparable to or exceed those reported in previous investigations examining lower-limb strength and mobility decline in older women [3,8,9,10,11,15,16], reinforcing the internal consistency of the associative findings observed in this cohort, without suggesting independent classification performance, diagnostic accuracy, or protocol validation.

Notably, MIHE showed strong associations with the chair-rise test (CRT), GS, and TUG outcomes. The large effect size observed for MIHE (d = 1.13) is aligned with prior literature indicating that proximal and lower limb strength often demonstrate stronger relationships with mobility outcomes than HG alone [5,6]. However, these findings should be interpreted as convergent associations within a cross-sectional framework, rather than as evidence of superior screening performance or validated classification capability. Moreover, the MIHE reflects hip extensor capacity and does not constitute a direct assessment of trunk muscle strength, despite its functional interaction with trunk stabilizers during upright and transitional tasks.

It is important to emphasize that the classification of probable sarcopenia in this study was based solely on the HG, as recommended by the EWGSOP2. Therefore, the observed differences in MIHE, MM, GS, TUG, CRT, and RMR should be interpreted as associations with this classification rather than as independent diagnostic or predictive indicators.

### 4.1. Anthropometric and Cardiometabolic Profile as a Substrate for Sarcopenia Risk

The anthropometric profile of the individuals participating in this study elucidates a demographic predominantly composed of older women residing within the community, who are primarily categorized as overweight, demonstrating a preserved measurement of calf circumference while simultaneously exhibiting elevated levels of central adiposity, as indicated by increased waist and abdominal circumferences. This trend is notably corroborated by extensive epidemiological studies conducted on Brazilian populations, which reveal a concerningly high incidence of both overweight and sarcopenic obesity in older women, even among those who maintain a socially active lifestyle, as referenced in various studies [17,18,19]. Central adiposity has been associated with poorer muscle quality, metabolic dysregulation, and functional decline in women; however, the strength and direction of these relationships vary across cohorts and study designs [20,21].

Nevertheless, when stratified according to HG, central adiposity markers (waist circumference, abdominal circumference, and waist-to-hip ratio) exhibited only small-to-moderate effect sizes between groups. In contrast, peripheral muscle-related anthropometric measures exhibited larger effect sizes, paralleling the magnitude of the differences observed for the functional tests in Table 5. This pattern suggests that peripheral muscle-related morphology may align more closely with HG-defined strength status than central adiposity in this sample, although the pilot design limits this inference.

This divergence between central and peripheral anthropometric indicators is consistent with previous findings, suggesting that appendicular muscle decline may be more tightly coupled to functional impairment than central adiposity per se [20,21]. However, given the pilot nature of this study, these morphological associations should be considered exploratory.

Stratified descriptive analyses indicated a higher proportion of cardiometabolic comorbidities in the group classified with probable sarcopenia (<16 kg), including hypertension, diabetes, dyslipidemia, and hypothyroidism. Because inferential comparisons were not performed for categorical variables, these differences are hypothesis-generating and may reflect broader health vulnerabilities rather than sarcopenia-specific mechanisms [22,23,24]. However, causality cannot be inferred from these cross-sectional data.

Additionally, the resting heart rate was higher in the probable sarcopenia group. Although the effect size was moderate and statistically significant, the literature remains inconclusive regarding whether an elevated resting HR reflects reduced cardiorespiratory fitness, altered autonomic balance, or generalized frailty processes [20,21]. Therefore, this finding should be interpreted cautiously.

### 4.2. Physical Activity Patterns and Their Functional Implications

Despite the undeniable existence of various cardiometabolic risk factors among the participants involved in this study, it was observed that these individuals consistently engaged in physical activity on a regular basis, predominantly favoring low-impact and group-oriented modalities such as water aerobics, gymnastics, and yoga, which are popular in this demographic. This exercise profile reflects the common preferences for physical activity among older women in Brazil and aligns with findings from numerous population-based studies that indicate a tendency for individuals to adopt socially oriented and moderate-intensity activities. However, regular participation does not necessarily preclude sarcopenia risk or functional vulnerability, particularly when training intensity and mechanical loading are limited [19,25].

However, while such modalities may confer cardiovascular, metabolic, and psychosocial benefits, they often involve moderate or low mechanical loading and may not provide sufficient stimulus to induce maximal neuromuscular adaptations, particularly in terms of strength and power development in older adults [25,26]. Evidence from resistance training trials consistently demonstrates that higher-intensity or progressive overload protocols are required to optimize gains in MS, neuromuscular function, and functional performance in aging populations. Therefore, although the physical activity habits observed in the present cohort are consistent with national patterns, they may not fully counteract the neuromuscular decline associated with aging and central adiposity, especially when maximal strength is considered.

The notable prevalence of light-to-moderate perceived exertion experienced by the participants, combined with the varied levels of adherence to these exercise regimens, may serve as a partial explanation for the observed clustering of functional performance and MS values that were found to be alarmingly close to the thresholds established by EWGSOP2. While it is undeniable that engaging in such low-impact activities contributes positively to the maintenance of mobility and balance among older adults, evidence from Brazilian cohorts indicates that increased adiposity and unfavorable body composition are significantly associated with reduced physical performance and muscle-specific strength in middle-aged and older women [27,28]. Taken together, these findings support the possibility that low-impact, socially oriented activities may maintain general function but may be insufficient to preserve proximal lower limb strength without progressive overload.

### 4.3. Functional Performance and EWGSOP2-Based Sarcopenia Screening

In alignment with the EWGSOP2 recommendations, a relevant proportion of participants presented SARC-F scores suggestive of an elevated sarcopenia risk [2]. Although the mean GS and TUG values were within community reference ranges, the large between-group effect sizes (GS d = 1.10; CRT d = 1.21; TUG d = 1.33) indicate that lower-limb–dependent tasks exhibited substantial differences according to HG-based stratification within this sample. These magnitudes are comparable to those reported in longitudinal and prospective studies linking poor TUG and CRT performance with fall risk and disability progression [10,11,12,29], supporting their ecological validity. Thus, functional tests may provide complementary information to HG within the EWGSOP2 screening, particularly for mobility-related impairment.

### 4.4. Maximal Isometric Hip Extension Strength as an Integrative Marker

One of the most significant and central findings derived from the comprehensive analysis conducted in this study is the notably pronounced reduction in MIHE, particularly among women classified as having probable sarcopenia based on the handgrip thresholds set forth by the EWGSOP2. MIHE primarily reflects the hip extensor force-generating capacity (predominantly gluteus maximus and synergistic musculature), which functionally interacts with trunk stabilizers during upright posture and transitional movements but does not directly assess trunk muscle strength. In quantitative terms, the between-group difference in MIHE was substantial (mean difference = 15.4 kg; 95% CI: 6.6–24.2), with a large effect size (d = 1.13). This magnitude is comparable to the effect sizes reported in studies linking lower limb strength to mobility limitation and frailty progression in older women [3,13,14]. Notably, the effect size observed for MIHE was similar to that detected for chair rise and gait speed, reinforcing the internal coherence of lower-limb–dependent measures. However, because classification was based exclusively on HS, MIHE should be interpreted as a strongly associated functional marker within this screening framework, rather than as an alternative classification parameter.

Accordingly, although the hip extensors and trunk musculature operate synergistically during sit-to-stand and gait tasks, MIHE is best interpreted as a proxy for proximal lower limb force production rather than a direct measurement of axial or trunk strength. This observation is broadly consistent with prior reports suggesting that lower limb strength may demonstrate stronger associations with mobility outcomes than handgrip strength alone [3,5,6].

Furthermore, the musculature responsible for hip extension and the trunk plays an essential and pivotal role in facilitating postural control, enabling gait propulsion, and assisting in the transitions from sitting to standing, which collectively renders MIHE a biomarker that is both biomechanically and functionally relevant to geriatric health [4,13]. Additionally, the robust and strong inverse association observed between MIHE and the time taken to rise from a chair in this study serves to further reinforce its ecological validity, thereby supporting the argument for its utilization as an integrative indicator of neuromuscular efficiency and functional independence among the elderly population.

### 4.5. Muscle Strength, Metabolic Rate, and Muscle Mass Interdependence

The significant and marked decline in the RMR, along with a noticeable reduction in muscle mass observed among participants likely suffering from sarcopenia, underscores the intricate relationship that exists between neuromuscular functionality and overall metabolic health, indicating the critical role of muscle tissue in maintaining metabolic homeostasis. The magnitude of the between-group differences further supports this interpretation. Muscle mass showed a large effect size (d = 0.96), while the resting metabolic rate demonstrated a similarly large effect (d = 0.97), with a mean difference of 141 kcal/day (95% CI: 47.2–235.0). These values are comparable to the differences reported in aging cohorts, where reductions in lean mass were associated with measurable declines in basal energy expenditure [1,30,31]. In older Brazilian women, dietary inadequacies and suboptimal protein intake may further contribute to the preservation challenges of lean mass and metabolic homeostasis [32,33], reinforcing the interconnected nature of neuromuscular and metabolic health.

Although RMR was estimated rather than directly measured via indirect calorimetry, the consistency of large effect sizes across MM, MIHE, and RMR supports internal coherence but does not establish directionality.

These findings are consistent with the well-established physiological role of skeletal muscle as a primary determinant of the resting metabolic rate, whereby reductions in muscle mass and proximal lower limb strength are associated with metabolic downregulation in older adults [30,31].

### 4.6. Functional Test Interrelationships and Sarcopenia Phenotyping

The interrelationships between CRT, GS, and TUG highlight the shared neuromuscular and balance demands of these tasks. The large effect sizes for CRT (d = 1.21), GS (d = 1.10), and particularly TUG (d = 1.33) indicate that lower-limb-dependent tasks demonstrated substantial between-group differences according to strength-based stratification within this sample. Importantly, this pattern reflects tasks that rely predominantly on proximal and lower-limb force production, including hip extensor capacity as captured by MIHE, rather than isolated trunk muscle performance. These magnitudes exceed the moderate correlations commonly reported between HG and mobility outcomes in older Brazilian women, suggesting that exclusive reliance on HG may underestimate lower-limb-specific impairments [5,6,12]. Nonetheless, HG remains a global indicator of muscle function, albeit with limited regional specificity.

Recent research indicates that exclusive reliance on HG may underestimate sarcopenia prevalence when alternative definitions incorporating lower limb strength or performance are considered [34,35,36]. Evidence suggests that upper limb strength does not consistently reflect lower limb neuromuscular capacity, with moderate associations reported between HG and knee extension or mobility outcomes [5,6]. In this context, MIHE should be interpreted as proximal lower limb extensor strength that contributes to postural control and transitions but does not constitute a direct measure of trunk or axial strength. The present findings are consistent with this dissociation, although confirmation would require longitudinal validation and alternative diagnostic frameworks [37].

### 4.7. Integrative Interpretation

The aggregate findings indicated a coherent pattern of concurrent reductions in proximal lower limb strength, functional performance, MM, and RMR. Although the MIHE interacts with trunk stabilizers during upright posture and transitional tasks, it should be interpreted as a marker of proximal lower limb force production rather than a direct assessment of trunk muscle strength. This integrative phenotype aligns with the view that sarcopenia in older women is effectively characterized by function-oriented evaluations emphasizing lower-limb capacity rather than isolated upper-limb strength measures [3,28,30,32,38]. Across domains, the consistency of large effect sizes (0.96 to 1.33 for functional and metabolic variables) supports internal coherence of the associative findings but should not be interpreted as evidence of superior screening performance or independent classification capability of lower-limb measures, since stratification was defined a priori by HG.

An additional dimension is the potential presence of inter-limb asymmetries and neuromuscular imbalances. In physically active older women with heterogeneous exercise backgrounds, sport-specific adaptations may induce unilateral discrepancies that are not detected by bilateral tests. Single-leg balance performance is a sensitive indicator of neuromuscular control and fall risk, and the absence of these assessments may limit the detection of subtle deficits. Future studies should incorporate asymmetry indices and standardized, single-leg balance protocols.

These findings reinforce the notion that reliance solely on the HG may underestimate neuromuscular deficits affecting mobility and fall risk. Incorporating assessments of proximal lower limb strength (such as MIHE) alongside metabolic markers may improve early detection strategies in primary care and community-based screening programs. However, such measures should be interpreted as complementary functional markers rather than direct substitutes for trunk-specific strength assessments or for established diagnostic criteria.

### 4.8. Study Limitations

This study is limited by its cross-sectional design, which precludes causal or temporal inference regarding the relationships between proximal lower limb strength (MIHE), gait speed, muscle mass, and resting metabolic rate. Accordingly, the findings should be interpreted as associations rather than predictions. The sample was small (n = 38) and drawn from a single community-based program of physically independent older women in Cabo Frio, which increases the risk of selection bias and limits generalizability to men, frailer adults, institutionalized populations, and other regions.

Additionally, because data collection was conducted within a community-based outreach initiative primarily focused on clinical screening and functional assessment, socioeconomic indicators such as educational level, income, and occupational background were not collected systematically. The absence of these variables limits the broader contextual characterization of the sample and restricts inferences regarding the social determinants of neuromuscular and metabolic outcomes.

Probable sarcopenia was operationally defined exclusively based on handgrip strength thresholds, in accordance with the EWGSOP2 recommendations. Although consistent with consensus guidelines, this approach does not constitute an independent diagnostic confirmation and may not fully capture the multidimensional complexity of sarcopenia.

Another limitation was the absence of unilateral strength assessments and single-leg balance evaluations. Therefore, inter-limb asymmetries and neuromuscular imbalances may have been undetected, particularly in physically active older women with heterogeneous exercise backgrounds.

Key exposures and covariates were also measured with constraints that could introduce misclassification or residual confounding, including reliance on BIA for muscle quantity estimation and the use of predominantly bivariate statistical analyses without multivariate adjustment.

Finally, no formal diagnostic accuracy analyses (e.g., ROC curves or predictive modeling) were conducted; therefore, the results should not be interpreted as establishing the diagnostic performance. Despite these limitations, the study provides preliminary evidence linking readily deployable functional measures to metabolic and body composition indicators in community-dwelling older women, supporting hypothesis generation and future longitudinal investigations.

## 5. Conclusions

In summary, this study demonstrated that maximal isometric hip extension strength (MIHE), a proxy of proximal lower limb extensor capacity, is strongly associated with functional performance, muscle mass, and resting metabolic rate in community-dwelling older women. Given the cross-sectional design and operational definition of probable sarcopenia based exclusively on handgrip strength according to the EWGSOP2 criteria, these findings should be interpreted as associative rather than causal.

While handgrip strength remains a practical and recommended screening tool within the EWGSOP2 framework, the assessment of proximal lower limb strength (e.g., MIHE) alongside mobility performance tests and metabolic indicators may provide a more comprehensive characterization of functional and metabolic impairments.

These measures should be interpreted as complementary functional markers rather than independent diagnostic tools or substitutes for the established criteria. Overall, the findings support a multidimensional assessment approach that expands functional evaluation while remaining consistent with the current consensus definitions of sarcopenia.

## Figures and Tables

**Table 1:** Anthropometric characteristics of the study participants overall and according to handgrip strength classification. Values are presented as mean ± standard deviation (SD) for the total sample (N = 38) and for groups defined by handgrip strength (<16 kg, n = 17; ≥16 kg, n = 21). Between-group comparisons are expressed as mean difference with 95% confidence intervals (CI), Cohen’s d effect size, and *p*-values.

Anthropometric Parameters	Mean ± SD (n = 38)	Mean ± SD		Mean Difference (95% CI)	Cohen’s d	*p*-Value
		<16 kg (n = 17)	>16 kg (n = 21)			
Age (y)	69.9 ± 6.6	70.4 ± 6.5	69.3 ± 5.9	−1.0 (−5.1–3.0)	0.18 (trivial)	0.61
Height (m)	1.6 ± 0.1	1.6 ± 0.1	1.6 ± 0.1	−0.02 (−0.07–0.03)	0.00 (trivial)	0.39
Body mass (kg)	64.5 ± 12.9	64.6 ± 12.5	67.9 ± 13.3	3.3 (−5.3–11.8)	0.25 (small)	0.44
BMI (kg/m^2^)	26.8 ± 5.0	26.7 ± 4.7	27.5 ± 2.7	0.8 (−1.7–3.2)	0.21 (small)	0.51
Waist circumference (cm)	85.7 ± 10.3	84.8 ± 7.8	88.0 ± 7.4	3.3 (−1.7–8.3)	0.41 (small)	0.19
Abdominal circumference (cm)	91.4 ± 11.2	90.9 ± 10.7	93.9 ± 7.9	3.1 (−3.0–9.2)	0.32 (small)	0.31
Hip circumference (cm)	103.1 ± 10.2	103.8 ± 9.9	103.9 ± 4.5	0.05 (−4.8–4.9)	0.01 (trivial)	0.98
Waste and hip ratio	0.8 ± 0.1	0.81 ± 0.06	0.84 ± 0.07	0.02 (−0.01–0.06)	0.46 (small)	0.21
Upper Arm Circumference (cm)	28.2 ± 3.2	25.8 ± 3.3	28.8 ± 2.4	2.9 (1.1–4.8)	1.08 (large)	0.002
Forearm Circumference (cm)	24.5 ± 2.3	23.6 ± 1.3	26.2 ± 1.3	2.6 (1.8–3.5)	2.02 (large)	0.0001
Calf Circumference (cm)	35.7 ± 3.8	35.0 ± 1.1	36.3 ± 0.7	1.2 (0.6–1.8)	1.57 (large)	0.0002

BMI: Body mass index.

**Table 2:** Cardiometabolic parameters, lifestyle characteristics and history of illness of the participants overall and stratified by handgrip strength (<16 kg vs. ≥16 kg). Continuous variables are presented as mean ± SD, with mean differences (95% CI), Cohen’s d, and *p*-values. Categorical variables are presented as N (%).

Cardiometabolic parameters	Mean ± SD (n = 38)	Mean ± SD		Mean Difference (95% CI)	Cohen’s d	*p*-Value
		<16 kg (n = 17)	>16 kg (n = 21)			
Blood glucose (mg/dL)	107.2 ± 46.4	116.3 ± 66.9	102.0 ± 16.1	−14.3 (−44.9–16.3)		0.34
HR rest (bpm)	72.3 ± 11.7	75.8 ± 7.5	69.9 ± 6.4	−5.9 (−10.5–−1.3)		0.01
DBP (mmHg)	80.1 ± 11.5	77.4 ± 9.4	77.2 ± 10.1	−0.14 (−6.6–6.4)		0.96
MAP (mmHg)	101.2 ± 11.8	95.7 ± 14.6	93.7 ± 15.1	−2.0 (−11.9–7.8)		0.67
SBP (mmHg)	143.4 ± 16.5	132.5 ± 35.3	126.6 ± 29.9	−5.9 (−27.3–15.6)		0.58
Lifestyle characteristics	n (%)	<16 kg (n = 17)	>16 kg (n = 21)			
Alcohol consumption	34 (89.5%)	25 (73.5%)	9 (26.5%)			
Smoker	4 (10.5%)	3 (75.0%)	1 (25.0%)			
History of illness	N (%) (n = 38)	<16 kg (n = 17)	>16 kg (n = 21)			
Cancer	1 (2.63%)	1 (5.8%)	0 (0%)			
Diabetes	7 (18.42%)	6 (35.3%)	1 (4.8%)			
Dyslipidemia	3 (7.89%)	3 (17.6%)	0 (0%)			
Hypertension	23 (60.53%)	14 (82.3%)	9 (42.9%)			
Hypothyroidism	4 (10.53%)	4 (23.5%)	0 (0%)			

Blood glucose and resting hemodynamic values, including SBP, DBP, MAP, and resting heart rate (HR), were expressed as mean ± SD. The frequencies of smoking and alcohol consumption, as well as the prevalence of comorbidities such as hypertension, diabetes, dyslipidemia, hypothyroidism, and cancer, are presented as absolute values and percentages.

**Table 3:** Physical activity modalities and habits of participants (N = 38), overall and stratified by handgrip-defined probable sarcopenia. Values are *expressed as n* (%) or mean ± SD. Groups: HG < 16 kg (*n* = 17) and HG ≥ 16 kg (*n* = 21). The modalities are not mutually exclusive.

Exercise Modalities	n (%) (n = 38)	<16 kg n (%) (n = 17)	>16 kg n (%) (n = 21)
Beauty Salon	5 (13.2)	4 (23.5%)	1 (4.8%)
Choir	3 (7.9)	3 (17.6%)	0 (0%)
Crafts	3 (7.9)	3 (17.6%)	0 (0%)
Dance	7 (18.4)	2 (11.8%)	5 (23.8%)
Global Postural Re-education (GPR)	2 (5.3)	2 (11.8%)	0 (0%)
Gymnastics	17 (44.7)	6 (35.3%)	11 (52.4%)
Memory Workshop	8 (21.1)	7 (41.2%)	1 (4.8%)
Physical Therapy and Acupuncture	8 (21.1)	5 (29.4%)	3 (14.3)
Pilates	5 (13.2)	3 (17.6%)	2 (9.5%)
Tai Chi Chuan	6 (15.8)	2 (11.8%)	4 (19.0%)
Water Aerobics	18 (47.4)	12 (70.5%	6 (28.6%)
Yoga	16 (42.1)	8 (47.1%)	8 (38.1%)
Physical activity habits	Mean ± SD	<16 kg n (%) (n = 17)	>16 kg n (%) (n = 21)
Duration (min)	45–60	45–60	45–60
Frequency (days/week)	3.3 ± 1.2	3.0 ± 1.2	4.5 ± 0.8 ****
Intensity (RPE)	3.4 ± 1.4	3.8 ± 1.6	3.1 ± 1.2
Time (mouths)	N (%)	<16 kg n (%) (n = 17)	>16 kg n (%) (n = 21)
0 to 3	11 (28.9)	7 (41.1%)	4 (19.0%)
3 to 6	3 (7.9)	2 (11.8%)	1 (4.8%)
6 to 9	5 (13.2)	3 (17.6%)	2 (9.5%)
9 to 12	7 (18.4)	3 (17.6%)	4 (19.0%)
>12	12 (31.6)	4 (23.5%)	8 (38.1%)

The number and percentage of individuals engaged in various forms of exercise, including water aerobics, gymnastics, yoga, Pilates, and Tai Chi Chuan. Physical activity habits were described by weekly frequency (days/week), perceived exertion intensity (rate of perceived exertion [RPE]), session duration, and duration of engagement (in months). These data provided an overview of the participants’ exercise behaviors and adherence to physical activity routines. **** *p* = 0.0001.

**Table 4:** Strength and functional performance tests were conducted among the study participants.

Strength and Physical Ability Tests	Mean ± SD
CRT (s for 5 rises)	12.0 ± 3.1
GS (m/s)	1.2 ± 0.4
HG (kg)	17.2 ± 5.5
MIHE (kg)	52.3 ± 15.3
TUG (s)	8.8 ± 1.6

Values are presented as mean ± SD values are deviation for handgrip strength, maximal isometric hip extension strength, gait speed, timed up-and-go, and chair rise test. These assessments reflect upper and lower limb strength, mobility, balance, and functional capacity in daily activities. Abbreviations: CRT, chair rise test; GS, gait speed; HG, handgrip; MIHE, maximal isometric hip extension; TUG, timed up-and-go.

**Table 5:** Study variables stratified by handgrip strength thresholds proposed by EWGSOP2. Data are presented as mean ± standard deviation (SD). Between-group differences were analyzed using independent-sample *t*-tests. Mean differences with 95% confidence intervals (CI) and standardized effect sizes (Cohen’s d) were reported.

Strength and Physical Ability Tests	Mean ± SD		Mean Difference (95% CI)	Cohen’s d	*p*-Value
	<16 kg	>16 kg			
CRT (s for 5 rises)	13.8 ± 3.5	10.5 ± 1.6	−3.3 (−5.0–−1.6)	1.21 (large)	0.0004
GS (m/s)	1.5 ± 0.3	1.0 ± 0.5	−0.4 (−0.7–−0.1)	1.10 (large)	0.002
HG (kg)	12.3 ± 3.6	21.3 ± 2.7	9.2 (7.1–11.2)	2.87 (large)	0.0001
MIHE (kg)	43.7 ± 15.7	59.1 ± 11.1	15.4 (6.6–24.2)	1.13 (large)	0.001
MM (kg)	37.6 ± 4.2	41.5 ± 3.9	3.9 (1.2–6.5)	0.96 (large)	0.005
RMR (kcal/day)	1008 ± 174	1149 ± 110	141.1 (47.2–235.0)	0.97 (large)	0.004
TUG (s)	9.8 ± 1.9	7.9 ± 0.7	−1.8 (−2.8–−0.9)	1.33 (very large)	0.0002

Maximal Isometric Hip Extension (MIHE), Gait Speed (GS), Timed Up and Go (TUG), Chair Rise Performance (CRT), Resting Metabolic Rate (RMR), and muscle mass (MM) in older women stratified by handgrip strength thresholds proposed by the EWGSOP2.

## Data Availability

The raw data supporting the conclusions of this article will be made available by the authors upon request.

## Abbreviations

The following abbreviations are used in this manuscript.

BIA	Bioelectrical Impedance Analysis
BMI	Body Mass Index
CRT	Chair Rise Test
DBP	Diastolic Blood Pressure
EWGSOP2	European Working Group on Sarcopenia in Older People 2
GS	Gait Speed
HG	Handgrip Strength
HR	Heart Rate
MAP	Mean Arterial Pressure
MIHE	Maximal Isometric Hip Extension
MM	Muscle Mass
MS	Muscle Strength
RMR	Resting Metabolic Rate
RPE	Rate of Perceived Exertion
SARC-F	Sarcopenia Risk Questionnaire
SBP	Systolic Blood Pressure
TUG	Timed Up-and-Go
WHO	World Health Organization
WHR	Waist-to-Hip Ratio
